# Comparison of Two-Step Transient Evoked Otoacoustic Emissions and One-Step Automated Auditory Brainstem Response for Universal Newborn Hearing Screening Programs in Remote Areas of China

**DOI:** 10.3389/fped.2021.655625

**Published:** 2021-05-14

**Authors:** Haibin Sheng, Qian Zhou, Qixuan Wang, Yun Yu, Lihua Liu, Meie Liang, Xueyan Zhou, Hao Wu, Xiangrong Tang, Zhiwu Huang

**Affiliations:** ^1^Department of Otolaryngology-Head and Neck Surgery, Shanghai Ninth People's Hospital, Shanghai Jiao Tong University School of Medicine, Shanghai, China; ^2^Ear Institute, Shanghai Jiao Tong University School of Medicine, Shanghai, China; ^3^Shanghai Key Laboratory of Translational Medicine on Ear and Nose Diseases, Shanghai, China; ^4^Department of Otolaryngology-Head and Neck Surgery, Liuzhou Maternal and Child Health Care Hospital, Liuzhou, China

**Keywords:** developing countries, neonatal hearing screening, screening time, otoacoustic emissions, automated auditory brainstem response

## Abstract

**Objective:** To compare the hearing screening results of two-step transient evoked otoacoustic emissions (TEOAE) and one-step automatic auditory brainstem response (AABR) in non-risk newborns, and to explore a more suitable hearing screening protocol for infants discharged within 48 h after birth in remote areas of China.

**Methods:** To analyze the age effect on pass rate for hearing screening, 2005 newborns were divided into three groups according to screening time after birth: <24, 24–48, and 48–72 h. All subjects received TEOAE + AABR test as first hearing screen, and those who failed in any test were rescreened with TEOAE + AABR at 6 weeks after birth. The first screening results of AABR and TEOAE were compared among the three groups. The results of two-step TEOAE screening and one-step AABR screening were compared for newborns who were discharged within 48 h. The time spent on screening was recorded for TEOAE and AABR.

**Results:** The pass rate of TEOAE and AABR increased significantly with the increase of first screening time (*P* < 0.05), and the false positive rate decreased significantly with the increase of first screening time (*P* < 0.05). The failure rate of first screening of AABR within 48 h was 7.31%, which was significantly lower than that of TEOAE (9.93%) (*P* < 0.05). The average time spent on AABR was 12.51 ± 6.36 min, which was significantly higher than that of TEOAE (4.05 ± 1.56 min, *P* < 0.05). The failure rate of TEOAE two-step screening was 1.59%, which was significantly lower than one-step AABR.

**Conclusions:** Compared with TEOAE, AABR screening within 48 h after birth can reduce the failure rate and false positive rate of first screening. However, compared with TEOAE two-step screening, one-step AABR screening has higher referral rate for audiological diagnosis. In remote areas of China, especially in hospitals with high delivery rate, one-step AABR screening is not feasible, and two-step TEOAE screening protocol is still applicable to UNHS screening as more and more infants discharged within 48 h after birth.

## Introduction

Due to the high incidence of neonatal hearing loss, congenital hearing loss has become the focus of health management department of countries all over the world. One ~3 among 1,000 healthy newborns and 2~4 among 100 high-risk neonates suffer from hearing loss as reported (1). The purpose of neonatal hearing screening is to reduce the negative effects of hearing loss on child's language, cognitive, social, emotional, and academic development through early detection (1).

The two important modes of neonatal hearing screening are high-risk neonatal hearing screening (HRNHS) and universal neonatal hearing screening (UNHS) (2). The first neonatal hearing screening program was developed in the 1960s to screen newborns at high risk of hearing loss (3). However, the importance of UNHS was recognized later as nearly half of newborns with congenital hearing loss are not from high-risk group (4). With the advancement and maturity of screening technology, UNHS has been established and implemented in many countries and regions. UNHS aims to screen all newborns no later than 1 month at age and provide comprehensive audiological evaluation for those who do not pass screening no later than 3 months of age. Infants diagnosed with hearing loss should receive appropriate intervention from health care and education professionals before 6 months of age (5, 6).

At present, two internationally recommended screening methods are otoacoustic emission (OAE) (7) and automatic auditory brainstem response (AABR) (8). Both methods will provide an objective result which shows “refer” or “pass” (2). OAE reflects the function of the cochlear outer hair cells, while ABR records the response from cochlea, auditory nerve and brainstem. For this reason, AABR will result in “refer” when screening infants with auditory neuropathy, whereas screening with OAEs will result in “pass” for the same baby (9). Therefore, AABR is recommended for high-risk newborns to detect auditory neuropathy (6). According to previous studies, the sensitivity of OAE was 90~95%, and the specificity was 89~91%; while the sensitivity of AABR was 100%, and the specificity was 96~98% (10–13). Compared with AABR screening, OAE is characterized by its simplicity and rapidity (2). Two-step OAE screening has been shown to be effective and is widely used in UNHS. However, there remains to be the problem of high false positive with OAE screening, especially for newborns within 48 h of birth (14). Despite being more expensive and taking longer to test, AABR has lower false positives and referral rates than OAE (7). One-step AABR screening has been reported to yield lower first-screening referral rates (1–4%) (15–18), which is lower than the recommended benchmark (<4%) for diagnostic hearing assessment (5). So it seems that one-step AABR screening protocol may be an effective screening model for UNHS. However, the referral rates for OAE and AABR screening in hospital-based settings in developing countries vary greatly (Philippines, TEOAE 10.3%, AABR 18.6%: Malaysia: DPOAE 49.9%, AABR 32.1%; South Africa: TEOAE 37.9%, AABR 16.7%) (2, 14, 19). As the improvement of obstetric technology, more and more babies will be discharged within 48 h after birth, especially in developing countries. Therefore, whether one-step AABR can be widely used in developing countries or even replace Two-step OAE is still questionable.

In this study, we compared the effectiveness of TEOAE and AABR as first screening tool at different time after birth in non-risk newborns. In order to find a better screening protocol suitable for the newborns discharged within 48 h after birth, we compared the practicability of two-step TEOAE and one-step AABR screening modes.

## Methods

### Participants

Participants in this study were healthy newborns without a history of NICU hospitalization born from October 2018 to February 2019 at the Liuzhou's Maternal and Child Health Care Hospital, which was a Tertiary Care Hospital in South China. A total of 2005 non-risk newborns were recruited into the study, of which 1015 (50.6%) were males and 990 (49.4%) were females. Demographical overview of the sample is provided in Table 1. All of the newborns were screened within 72 h after birth. The including criteria were newborns with gestational age ≥37 weeks and body weight ≥2,500 g. Newborns with any risk factors for hearing loss were excluded. The withdrawal criteria included neonatal or infantile mortality, parental refusal for hearing screening and foreigners. To further analyze the influence of age on the pass rate of hearing screening, the newborns were divided into three groups according to screening time after birth: <24, 24–48, and 48–72 h (Table 1). The study was approved by the Ethics Committee of Maternal and Child Health Care Hospital, and the parents of all newborns in the study signed written informed consent.

**Table 1:** Demographic data for 2005 non-risk infants.

**Variables**	***N*(%)**
**Gender**	
Male	1,015 (50.6%)
Female	990 (49.4%)
**Age at first screening for TEOAE and AABR**	
<24 h	673 (33.6%)
24–48 h	667 (33.3%)
48–72 h	665 (33.1%)
“Fail” first screening	189 (9.4%)
“Fail” second screening	28 (1.4%)
**Hearing status**	
No hearing loss	1,951 (97.3%)
Hearing loss	12 (0.6%)
Unilateral	4 (0.2%)
Bilateral	8 (0.4%)
Unknown status (defaulted second screening)	42 (2.1%)

### Screening and Diagnostic Procedures

Both TEOAE and AABR test were conducted as the first hearing screening protocol for all neonates before discharge from hospital. The ambient noise level of the maternity ward where the first screening was conducted was below 40 dB SPL. The time spent on TEOAE and AABR tests was recorded separately. Infants are screened while they are asleep or quiet. The ear canal of infants should be cleaned before screening. If both TEOAE and AABR test pass, it is defined as passing the hearing screening; if any one of them fails, it is defined as hearing screening failure.

**Figure 1:**
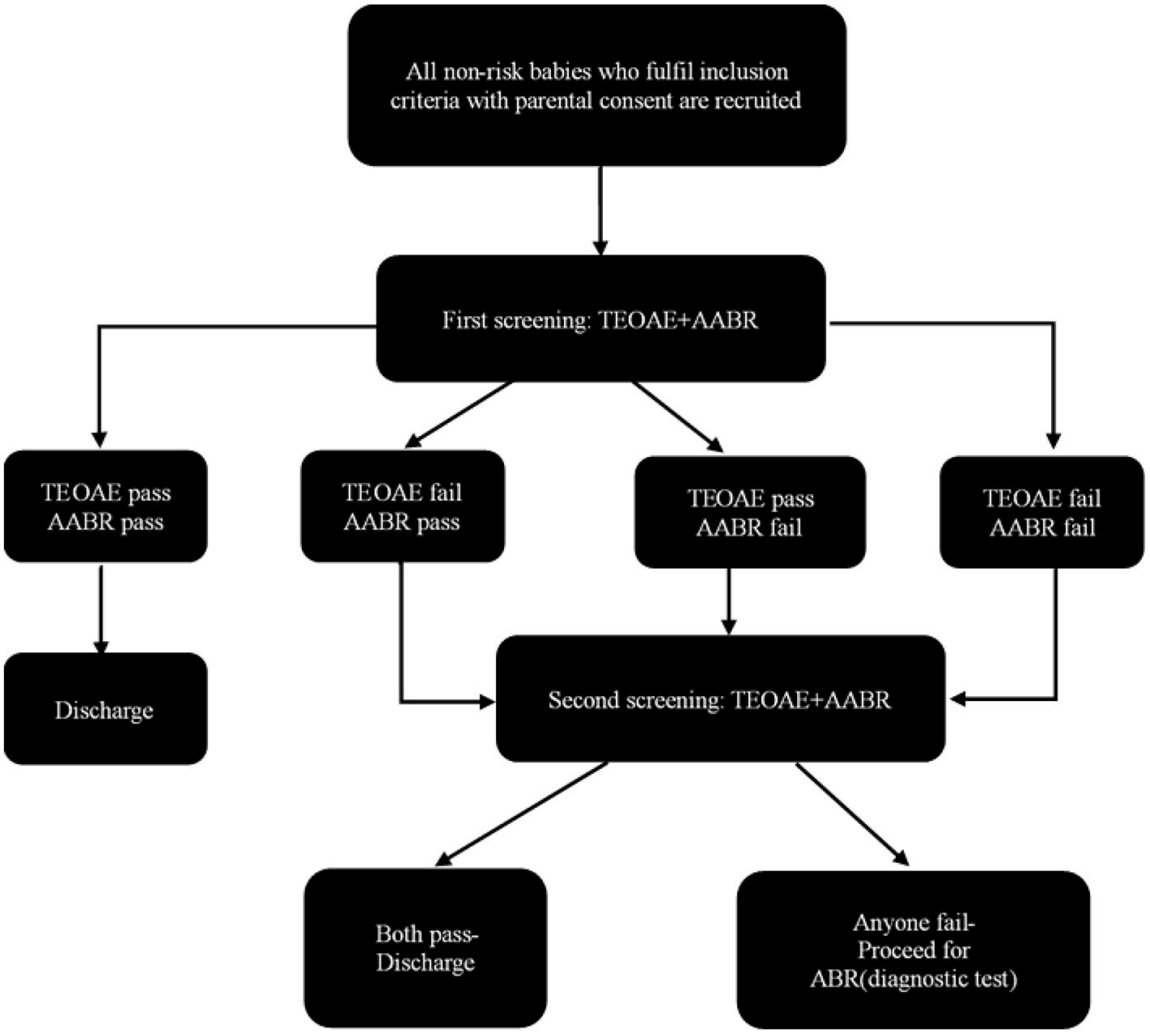
Screening protocol.

The rescreening was conducted about 42 days later in a room with background noise <40 dB SPL. Both TEOAE and AABR test were performed. Passing standard was the same as primary screening. Infants who failed rescreening were scheduled for diagnostic evaluation at hearing diagnosis center within 3 months after birth. The hearing diagnostic methods include 1,000Hz acoustic immittance test, diagnostic DPOAE and ABR test. The hearing rescreening and hearing diagnostic tests were performed by professional audiologists and otolaryngologists. More details were showed in Figure 1.

### Material and Apparatus

The TEOAE and AABR screening test were conducted with AccuScreen hearing-screening Instrument (Madsen-GN Otometrics, Taastrup, Denmark). For TEOAE test, the stimulus sound is a non-linear click sequence with a frequency range of 1.5 to 4 kHz, and its intensity is about 70–84 dB SPL. According to the response amplitude and signal-to-noise ratio, the device automatically determined whether the test results were “pass” or “refer.” For AABR test, three disposable electrodes were placed onto the baby's forehead, cheek, and neck before testing. The test can start only when the impedance between any two electrodes is no more than 12 kΩ. The stimulatory signals were clicks at an intensity of 35 dB nHL with a rate of approximately 55 Hz. The device automatically produced a result of “pass” or “refer” according to the infant's recorded reaction.

The Auditory Evoked Potentials system and ICS Chartr EP 200 instrument (Natus, Mundelein, IL, USA) were used for diagnostic ABR testing. The stimulatory signals were clicks with alternating polarity at a pulse width of 0.1 ms with a repetition rate of 19.3 ms. Disposable electrodes were attached to the forehead, ipsilateral mastoid and contralateral mastoid as recording electrodes, reference electrodes and ground electrodes, respectively. The impedance between any two electrodes was no more than 5 kΩ, and the bandpass filter was set at 100–3,000 Hz. For the air-conducted click ABR, a wave V reaction threshold of ≤ 30 dB nHL is regarded as normal ABR threshold, and a response threshold of ≥35 dB nHL was considered to be abnormal (20). Infants with hearing loss were further tested with a bone-conducted click stimulate to determine the type of hearing loss (conductive, sensorineural or mixed). A frequency-specific (toneburst 500, 1,000, 2,000, and 4,000Hz) ABR evaluation was conducted to determine the degree of hearing loss. The average hearing threshold (dB HL) was evaluated according to the threshold of toneburst 500, 1,000, 2,000, and 4,000Hz ABR. Hearing loss was classified into mild (26~40 dB), moderate (41 ~ 60 dB), severe (61~80 dB) and extremely severe(>80 dB).

### Statistical Methods

Statistical analyses were performed using SPSS 22.0. Descriptive analysis was used to show basic trends in demographical variables and screening results. Chi-square test and paired *T*-test were used to determine significance of differences between the two screening technologies. A *p* < 0.05 was taken to be a significant difference.

## Results

One hundred eighty nine (9.4%) failed TEOAE or AABR tests on one or both ears for the first screening among the 2005 newborns recruited for the study (Table 1). Thirty five infants did not participate in the second screening. A rescreening was performed in 154 infants. Twenty eight (1.4%) infants failed the TEOAE or AABR tests on one or both ears and were recommended for audiological diagnosis. Seven newborns were not diagnosed because of their parents' refusal. Twelve (0.6%) newborns were diagnosed with hearing loss. Among them, 4 (33.3%) were unilateral hearing loss and 8 (66.7%) were bilateral hearing loss. The degree of hearing loss was 3 (25%) mild hearing loss, 4 (33.3%) moderate hearing loss, 4 (33.3%) severe hearing loss, and 1 (8.3%) extremely severe hearing loss. For hearing loss types, there were 4 (33.3%) conductive hearing loss, 6 (50%) sensorineural hearing loss, and 2 (16.7%) mixed hearing loss.

The first screening pass rate of AABR test (93.77%) was higher than TEOAE test (91.22%) (Table 2). The pass rates of TEOAE and AABR tests among three groups were compared (Table 3). With the increase of age, the pass rates of TEOAE and AABR improved significantly (*p* < 0.05). The highest pass rate for both TEOAE and AABR were between the age of 48–72 h.

**Table 2:** The pass and failure rates (n/%) of first screening in 2005 neonates during the first screening.

	**Passed AABR**	**Failed AABR**	**Total**
Pass TEOAE	1,817 (90.63%)	12 (0.59%)	1,829 (91.22%)
Failed TEOAE	63 (3.14%)	113 (5.64%)	176 (8.18%)
Total	1,880 (93.77%)	125 (6.23%)	2,005 (100%)

**Table 3:** The pass rates (n/%) of first TEOAE and AABR screening in 2005 neonates as a function of age.

	**Passed TEOAE**	**Passed AABR**
<24 h	600 (89.15%)	615 (91.38%)
24–48 h	607 (91.00%)	627 (94.00%)
48–72 h	622 (93.53%)	638 (95.94%)
Total	1,829 (91.22%)	1,880 (93.77%)
Chi-square test	*p* = 0.018 <0.05	*p* = 0.002 <0.01

The prevalence of hearing loss among three groups which were screened at different time after birth were compared (Table 4). No significant statistical difference was found among the three groups (χ^2^ = 2.007, *p* = 0.367).

**Table 4:** Prevalence of hearing loss of newborns screened at different time.

	**Prevalence of hearing loss**
<24 h	4 (0.59%)
24–48 h	4 (0.90%)
48–72 h	2 (0.30%)
Chi-square test	*p* > 0.05

The failure rates and false-positive rates of TEOAE and AABR tests among three groups were compared (Table 5). Both TEOAE and AABR failure rate and false-positive rate decreased significantly with increasing age (*p* < 0.05). The group screened between the age of 48–72 h had the lowest failure rate and false-positive rate for both TEOAE and AABR tests. For each group, the failure rate and false-positive rate with AABR were significantly lower than that with TEOAE (*p* < 0.05).

**Table 5:** The failure rate and false-positive rate of the first screening at different time.

	**Failure rate**		**False-positive rate**	
	**AABR**	**TEOAE**	**AABR**	**TEOAE**
<24 h	8.62	10.95	8.22	10.31
24–48 h	6.00	9.00	5.14	8.17
48–72 h	4.06	6.47	4.07	6.18

**Figure 2:**
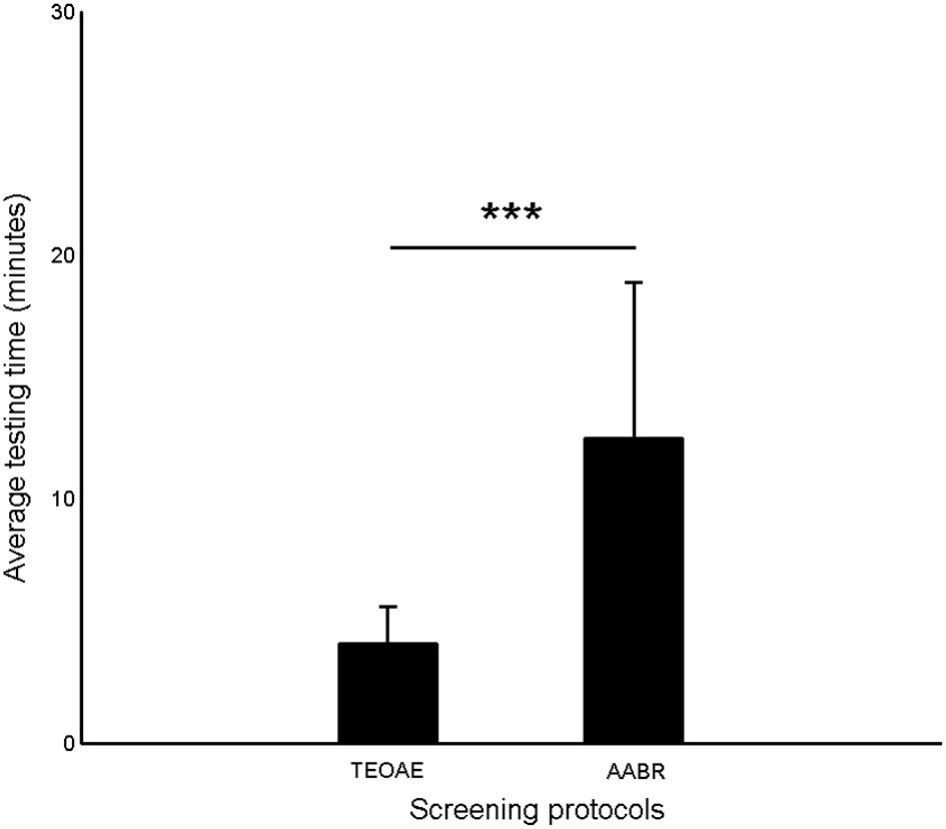
The average testing time of TEOAE and AABR. Error bars indicate one standard deviation (^***^p < 0.001, Paired *T*-test).

Figure 2 shows the average testing time of TEOAE and AABR. It was obvious that AABR test (12.51 ± 6.36 min) cost more time than TEOAE (4.05 ± 1.56 min) (*p* < 0.001).

To compare the screening effectiveness of two-step TEOAE and one-step AABR for infants discharged within 48 h, we listed the results of the two modes, respectively (Table 6). The differences between the two screening protocols are statistically significant (*p* < 0.05) in all the variables chosen, except for the number of hearing loss cases diagnosed and positive hearing loss predictive value with each procedure. The failure rate of the first screening with AABR was significantly lower than that with TEOAE (*p* < 0.05), but higher than the failure rate of the second screening with TEOAE (*p* < 0.01). The rate of false positives for the first screening phase was 6.57% with AABR, which was significantly lower than 9.18% with TEOAE (*p* < 0.05), and was significantly higher than 0.83% for the false positive rates of second screening with TEOAE (*p* < 0.01).

**Table 6:** Results of hearing screening with TEOAE and AABR of neonates discharged within 48 h after birth.

	**Two-step TEOAE**			**One-step AABR**	***p*-value**
	***N* (infants)**	**%**	***N* (infants)**	**%**	
Number of newborns	1,340		1,340		
“Fail” first screening	133/1,340	9.93	98/1,340	7.31	0.016
No-show for second screening	18/133	13.53	–	–	
“Fail” second screening	21/1,322	1.59	–	–	–
Referred for ENT diagnosis	21		98		
Now-show for diagnosis	5/1,322	0.37	24/1,340	1.79	
Hearing loss	10/1,322	0.76	10/1,340	0.75	0.976
**False-positive rates**					
First screening	123/1,340	9.18	88/1,340	6.57	0.012
Second screening	11/1,322	0.83	–	–	–

## Discussion

Hearing loss in early childhood can impede the children's speech, language and cognitive development, causing adverse effects on social, emotional and academic development with increasing social cost (2). UNHS has been implemented in countries all over the world to detect neonates with congenital hearing loss early and provide appropriate interventions in time. Therefore, it is extremely important to find a convenient and effective screening protocol to identify precisely all newborns with hearing loss (21). With the improvement of obstetric technology, more and more babies will be discharged within 48 h after birth. This study mainly explored the effectiveness of TEOAE and AABR as first screening tool at different time after birth in non-risk newborns and compared the practicability of two-step TEOAE and one-step AABR screening methods for infants discharged with 48 h after birth.

The first screening pass rate of AABR was significantly higher than that of OAE, with a difference of 2.6%, which means that 26 more babies per 1,000 newborns will not be able to pass the first screening with OAE compared to AABR, which demonstrated lower AABR referral rates that is similar with other reports (2, 14, 19). OAE screening records the sound energy emitted by the active movement of the inner ear and outer hair cells to evaluate cochlear function (22). The result of OAE screening is susceptible to the function of middle ear and external ear, especially the former (23). AABR records the electrical response of the auditory brainstem after acoustic stimulation, which is much less affected by middle and external ear functioning than TEOAE. This may explain for the lower positive rate of AABR screening compared with TEOAE.

For prevalence of hearing loss, we found that age had no significant effect on it, indicating that the first screening time will not affect the final diagnosis rate of hearing loss. In addition, our research shows that the first screening pass rate of TEOAE and AABR is significantly related to the screening time, and the pass rate increases as the newborns get older. As Benito Orejas said, the reason maybe that debris in the ear canal significantly reduced and the transient middle ear effusion resolved on the second day after birth (18). For the same reason, Gabbard and Doyle recommended OAE screening 48 h after birth (14, 24). In addition, the false positive rate of initial screening with TEOAE in each group is significantly higher than that of AABR, indicating that AABR is less affected by external and middle ear conditions, which seems to be a better choice for those countries where mother and babies can be discharged within 48 h after birth.

However, in the selection of neonatal hearing screening program, we should consider the sensitivity and accuracy of screening tool, as well as its feasibility. We found the average test time of AABR is about three times that of TEOAE. The OAE test is usually faster than the AABR test, even though the time it takes may vary depending on the machine used (2, 14). It is a challenge for hospitals with high delivery rate to perform AABR screening for every neonate. On the other hand, the hearing screening program need to adapt to the current situation of shorter discharge time after birth. Therefore, we compared the applicability of two-step TEOAE and AABR alone screening programs within 48 h after birth. The first screening failure rate (9.93%) and the rescreening failure rate (1.59%) of TEOAE test were similar to those of some other reports (18, 25), which meet the referral rate requirements for hearing diagnosis (5). The results showed that two-step TEOAE screening was suitable for newborn hearing screening within 48 h after birth. Compared with TEOAE, the failure rate (7.31%) of one-step AABR screening was significantly lower. However, this result was higher than 2.6% in Benito-Orejas et al. (18). The reason may be that the technicians in remote areas of developing countries are not proficient enough. In addition, due to high delivery rate, a large number of newborns need to be screened so it is impossible to wait until the neonates are well-asleep before testing. The result of AABR screening will be more susceptible to the newborn's status given that AABR screening costs more time than TEOAE. Therefore, the referral rate of one-step AABR was too high, which could not meet the referral requirements for diagnostic audiology examinations (4%). This further demonstrated the importance of audiology education and training, which helps to reduce the false positive rate of hearing screening and reduce the economic burden of hearing diagnosis. Although AABR has the advantages of lower failure rate and screening for auditory neuropathy compared with OAE, it has higher requirements for the testing environment, the testing status of the newborn, the testing time, and the testing personnel (7, 9). It is necessary to train and assess the qualifications of technicians before performing AABR test. For developing countries, especially China, due to the large differences in medical resources between regions, AABR technology needs to be verified on a large scale before its application. In a word, at present hearing screening protocol of one-step AABR cannot replace two-step TEOAE in terms of time cost and referral rate.

## Conclusion

From above all, we can conclude that the failure rate and false-positive rate of first hearing screening decreased significantly with increasing screening time. Though AABR has lower failure rate and false positive rate of first screening than TEOAE, one-step AABR screening can't meet the referral rate requirements for diagnostic audiology evaluation due to its relatively high failure rate. For Developing countries or regions, especially in hospitals with a high delivery rate, two-step TEOAE screening protocol is still applicable to UNHS for newborns discharged within 48 h after birth.

## Data Availability Statement

The raw data supporting the conclusions of this article will be made available by the authors, without undue reservation.

## Ethics Statement

The studies involving human participants were reviewed and approved by the Ethics Committee of Liu Zhou Maternal and Child Health Care Hospital. Written informed consent to participate in this study was provided by the participants' legal guardian/next of kin.

## Author Contributions

HS, QZ, and QW were responsible for the design and development of the study. YY, LL, ML, and XZ recruited participants and collected data. HS and QZ complete the first draft of the manuscript with help from XT, ZH, and HW. Data analysis was mainly achieved by QZ. All authors contributed to the manuscript revisions, and read and approved the submitted version of the manuscript.

## Conflict of Interest

The authors declare that the research was conducted in the absence of any commercial or financial relationships that could be construed as a potential conflict of interest.
